# Saunders’s Medical Hand-Atlases

**Published:** 1902-07

**Authors:** 


Saunders's Medical Hand-Atlases. Atlas and Epitomé of Operative Surgery. By Dr. Otto Zuckerkandl, Privadocent in the University of Vienna. From the second revised and enlarged German edition. Edited, with additions, by J. Chalmers DaCosta, M. D., Professor of the principles of Surgery and Clinical Surgery, Jefferson Medical College, Philadelphia, etc. Second edition, thoroughly revised and greatly enlarged. With 40 colored plates, 278 text illustrations and 410 pages of text. Philadelphia and London: W. B. Saunders & Co. 1902. [Cloth, $3.50 net.]

When this work was first published it was greeted generally as an excellent and practically helpful book. The second edition which has now been put out is an improvement on the first, in that it is larger, and has been brought strictly up to date, and it is apparent even to a most casual observer that the work of revision has not been hastily done, but thoroughly and exhaustively, and everywhere from cover to cover are evidences of careful scrutiny. The illustrations have been increased in number and in addition to being beautifully artistic are scientifically correct. Particularly is this so of those colored plates which are intended to show operative methods.

A number of the chapters have been rewritten and all the newer operations are described minutely and clearly. There is no volume published which can so nearly take the place of clinical instruction as this. N.W.W.

